# Light-Induced Charge Separation in Photosystem I from Different Biological Species Characterized by Multifrequency Electron Paramagnetic Resonance Spectroscopy

**DOI:** 10.3390/ijms25158188

**Published:** 2024-07-26

**Authors:** Jasleen K. Bindra, Tirupathi Malavath, Mandefro Y. Teferi, Moritz Kretzschmar, Jan Kern, Jens Niklas, Lisa M. Utschig, Oleg G. Poluektov

**Affiliations:** 1 Argonne National Laboratory, Chemical Sciences and Engineering Division, 9700 South Cass Avenue, Lemont, IL 60439, USA; j.bindra@anl.gov (J.K.B.); tmalavath05@gmail.com (T.M.); mteferi@anl.gov (M.Y.T.); jniklas@anl.gov (J.N.); 2 Lawrence Berkeley National Laboratory, Molecular Biophysics and Integrated Bioimaging Division, Berkeley, CA 94720, USA; mkretzschmar@lbl.gov (M.K.); jfkern@lbl.gov (J.K.)

**Keywords:** photosynthesis, electron paramagnetic resonance spectroscopy, photosystem I

## Abstract

Photosystem I (PSI) serves as a model system for studying fundamental processes such as electron transfer (ET) and energy conversion, which are not only central to photosynthesis but also have broader implications for bioenergy production and biomimetic device design. In this study, we employed electron paramagnetic resonance (EPR) spectroscopy to investigate key light-induced charge separation steps in PSI isolated from several green algal and cyanobacterial species. Following photoexcitation, rapid sequential ET occurs through either of two quasi-symmetric branches of donor/acceptor cofactors embedded within the protein core, termed the A and B branches. Using high-frequency (130 GHz) time-resolved EPR (TR-EPR) and deuteration techniques to enhance spectral resolution, we observed that at low temperatures prokaryotic PSI exhibits reversible ET in the A branch and irreversible ET in the B branch, while PSI from eukaryotic counterparts displays either reversible ET in both branches or exclusively in the B branch. Furthermore, we observed a notable correlation between low-temperature charge separation to the terminal [4Fe-4S] clusters of PSI, termed F_A_ and F_B_, as reflected in the measured F_A_/F_B_ ratio. These findings enhance our understanding of the mechanistic diversity of PSI’s ET across different species and underscore the importance of experimental design in resolving these differences. Though further research is necessary to elucidate the underlying mechanisms and the evolutionary significance of these variations in PSI charge separation, this study sets the stage for future investigations into the complex interplay between protein structure, ET pathways, and the environmental adaptations of photosynthetic organisms.

## 1. Introduction

Natural photosynthetic conversion of light energy to chemical energy is essential to all life on Earth. The primary energy conversion reactions involve photoinitiated rapid sequential electron transfer (ET) steps that result in the formation of a stabilized, long-lived charge-separated state across a biological membrane. These reactions occur in large, integral membrane protein complexes called reaction centers (RCs) [1,2]. The oxygenic photosynthesis that occurs in plants, algae, and cyanobacteria utilizes two types of RCs, Photosystem II (PSII) and Photosystem I (PSI) [3,4,5,6]. PSII catalyzes the light-driven oxidation of water whereas PSI catalyzes light-driven transmembrane electron transfer from reduced plastocyanin to oxidized ferredoxin, which subsequently shuttles the reducing equivalents from PSI to various metabolic pathways including CO_2_ fixation. Each RC has at its core a network of molecular cofactors embedded within a protein matrix which specifically positions each cofactor in optimal geometry and distance along efficient electron transfer pathways.

**Figure 1:**
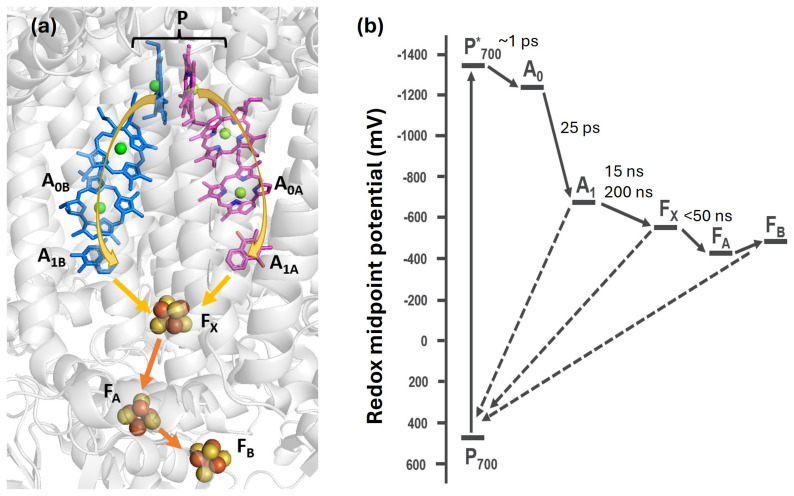
(**a**) Schematic structure and ET pathways in cyanobacterial Photosystem I (PDB ID 1JB0). Following photoexcitation, the excited primary donor, P*_700_, becomes oxidized, transferring one electron to one of two almost identical chains of electron transfer cofactors (chlorophyll A_0_ and phylloquinone A_1_) and converging at the three [4Fe-4S] clusters, F_X_, F_A_, and F_B_. Photoinduced ET in PSI is bidirectional at ambient temperature, proceeding through both the A and B branches of cofactors as indicated by arrows. (**b**) Energy diagram and time constants of forward electron transfer reactions in PSI. Information was taken from refs. [12,13].

The first high-resolution structure of PSI was resolved by X-ray crystallography for the thermophilic cyanobacterium, *Thermosynechococcus elongatus* (previously named *Synechococcus elongatus* and recently renamed to *T. vestitus*) [7,8,9]. Two large subunits (PsaA and PsaB) form the heterodimeric core of PSI, which has the same overall structure for different biological species. These two subunits bind the antenna pigments (about 100 chlorophyll and 20 carotenoid molecules) and the redox cofactors involved in the primary ET processes. Two branches of ET cofactors are arranged in a pseudo two-fold symmetry and are referred to as the A and B branches (Figure 1). Following photoexcitation, the primary electron donor, P_700_ (a dimer of chlorophyll molecules), becomes oxidized, transferring one electron to one of the two nearly identical ET branches that each contain two additional chlorophyll (Chl) molecules (an accessory Chl and the primary acceptor A_0_), and one phylloquinone (Vitamin K_1_; termed A_1_ or secondary acceptor). From reduced A_1_^−^, the electron is transferred to [4Fe-4S] cluster F_X_, located at the interface between PsaA and PsaB, and then to the terminal electron acceptors, F_A_ and F_B_, two [4Fe-4S] clusters housed within an extrinsic protein subunit, PsaC, located on the stromal side of PSI [3,6,7,10,11,12,13]. Note, that the very first ultrafast steps of charge separation, including the involvement of the accessory Chls, have been extensively investigated by ultrafast optical spectroscopy and different models of the initial steps were proposed [3,14,15,16,17]. More recently, high-resolution crystal and cryo EM structures of PSI (and PSI-LHCI supercomplexes) from different organisms, like higher plants [18,19], green algae [19,20,21], and mesophilic cyanobacterium *Synechocystis* sp. PCC 6803 [22], have been determined, allowing structural comparison through an evolutionary lens. Photosynthetic organisms contain the PSI complex in different, but related forms tuned for efficient charge separation with near unity quantum yield.

For a long time, the ET in PSI was presumed to be unidirectional, i.e., only along the A branch by analogy to the Type II purple non-sulfur bacterial photosynthetic RC and Photosystem II, despite no different functionality of the quinones A_1A_ and A_1B_. According to the primary paradigm, ET in PSI occurs only along the A branch and is blocked at low temperatures beyond A_1A_ quinone. This model is in agreement with our previous time-resolved (TR) electron paramagnetic resonance (EPR) studies of PSI from thermophilic *Synechococcus lividus* and mesophilic *Synechococcus leopoliensis*, where only one type of short-lived radical pair (RP), namely, P_700_^+^A_1A_^−^, was observed [23,24], and with a number of time-resolved EPR studies [25,26,27,28]. However, EPR spectra from PSI illuminated at low temperature always reveal the generation of photoinduced strong stable signals of P_700_^+^ and reduced [4Fe-4S] clusters, which is direct evidence that ET from P_700_ to F_X_, F_A_, and F_B_ occurs in a fraction of PSI complexes at low temperature. This was originally explained within the unidirectional model as ET occurring via the A branch in partially damaged RCs or RCs in a different conformational state [23]. As an alternative explanation, it was suggested that this ET does not occur via A branch but through the B branch, where the electrons do not stop at the quinone as a RP P_700_^+^A_1B_^−^ even at low temperatures, but rather proceeds further to F_X_, F_A_, and F_B_. At low temperature, the [4Fe-4S] clusters F_A_ and F_B_ act as deep traps for electrons, and thus, electron recombination to the oxidized primary donor P_700_^+^ is largely suppressed. Therefore, at low temperatures, where most TR-EPR experiments are performed, the only observable transient RP is from the A branch of PSI. Because the charge-separated state P_700_^+^A_1A_^−^ is short lived (≤200 μs) due to charge recombination, this ET is often called “reversible” or “cyclic”. In analogy, the ET is called “irreversible” if the electron can proceed beyond A_1_ and becomes trapped on [4Fe-4S] clusters at low temperatures [11,12,29,30].

High-frequency/high-field D-band (130 GHz) TR-EPR experiments enabled the first direct detection of the transient radical pairs P_700_^+^A_1A_^−^ and P_700_^+^A_1B_^−^ in cyanobacterial PSI, which are clearly resolved at this frequency [23,27]. The geometric parameters of the two distinct donor/acceptor pairs correspond to the charge-separated states along the A and B branches and are in excellent agreement with the X-ray crystal structure of PSI [23]. These experiments clearly demonstrated bidirectional ET at low temperature (≤100 K) under strongly reducing conditions for the thermophilic *S. lividus* PSI. Another crucial set of experiments that examined the involvement of the A and B branches in ET was performed on PSI from mesophilic *S. leopoliensis* where the [4Fe-4S] clusters F_X_, F_A_, and F_B_ were removed to prevent forward ET beyond the quinones. TR-EPR spectra of these biochemically modified PSI complexes at 100 K are comprised of two overlapping signals: one from the transient radical pair in the A branch, P_700_^+^A_1A_^−^, and another from the transient pair formed in the B branch, P_700_^+^A_1B_^−^, with an almost equal ratio of ET through A and B branches [24].

These results were possible due to two essential features of the experimental design: deuteration of PSI, which improves the spectral resolution by decreasing the EPR line width, and the high resolution afforded by high-frequency (HF) TR-EPR at 130 GHz/4.6 T. The deuteration of photosynthetic microorganisms by adaption and growth of bacteria, cyanobacteria, and algae in heavy water (99.6% D_2_O) was pioneered over 60 years ago [31,32,33]. EPR studies of RCs isolated from both protonated and deuterated non-sulfur purple bacteria and cyanobacteria have helped resolve the cofactors and local protein environments involved in light-induced charge separation events [23,25,34,35,36,37,38,39]. In contrast, advanced HF EPR studies of green algal PSI are lacking. Now, we report the first EPR studies of deuterated PSI isolated from the green alga, *Chlorella vulgaris* and *Scenedesmus obliquus,* grown in 99.6% D_2_O. This allows us to obtain high-resolution TR-EPR of the transient radical pair P_700_^+^A_1_^−^ at D-band (130 GHz) and interrogate ET to the terminal [4Fe-4S] acceptors with X-band EPR studies.

The bidirectional nature of ET in PSI at ambient temperature is nowadays well accepted [23,24,30,34,37,39,40,41,42,43]. At the same time, a very important question remains: how does electron transfer differ from species to species and is there any relation to the electronic properties of [4Fe-4S] clusters? While there have been investigations of PSI isolated from various species, they typically used different methodologies and approaches which make direct comparisons challenging. In this publication, we report a comparative study of isolated PSI from five biological species: *S. lividus*, *S. leopoliensis*, *T. elongatus*, *Chlorella vulgaris*, and *Scenedesmus obliquus*. The former three species are either mesophilic or thermophilic cyanobacteria and thus prokaryotes, while the latter two are green algae and thus eukaryotes (like higher plants). RC charge separation is sensitive to dynamic protein conformational substates and local heterogeneous protein environments that surround the cofactors [34,44]. The comparison of species-dependent spectral signatures thus can provide insight into evolutionary adjustments of PSI protein matrices and charge-separation to different environmental conditions and provide valuable insight into the mechanisms employed by nature to fine tune ET in RCs.

## 2. Results and Discussion

### 2.1. Spin Correlated Radical Pair (SCRP)

To obtain spectral signatures of PSI ET pathways from different species, we performed time-resolved EPR (TR-EPR) experiments at high magnetic fields [45,46]. Optical excitation of PSI initiates ET, leading to the generation of one or more sequential radical-ion pairs (RPs), including the so-called secondary pair P_700_^+^A_1_^−^ (earlier RPs are too short-lived to be detected by EPR). The weakly interacting spins of the radical pair are initially entangled or correlated and are known as spin-correlated radical pairs (SCRPs) [25,34,35,47,48,49]. Since the SCRPs are created by rapid ET from the photoexcited singlet state of the primary donor P_700_, initially only the states in the four-level system that have singlet characters are populated [47,50]. This strong non-Boltzmann electron spin polarization results in line shapes different from radical pair (RP) spectra in thermal equilibrium, creating a series of alternating emissive and absorptive lines (“antiphase doublets”). These SCRPs are exceptionally sensitive to weak magnetic interactions, structure, and heterogeneous local protein environments and thus can be used as highly sensitive sensors for any changes, e.g., in mutual orientation of the paramagnetic cofactors, P_700_^+^ and A_1_^–^ [26,51,52,53,54]. The line shape of the SCRP is especially informative when recorded with high spectral resolution at HF EPR.

**Figure 2:**
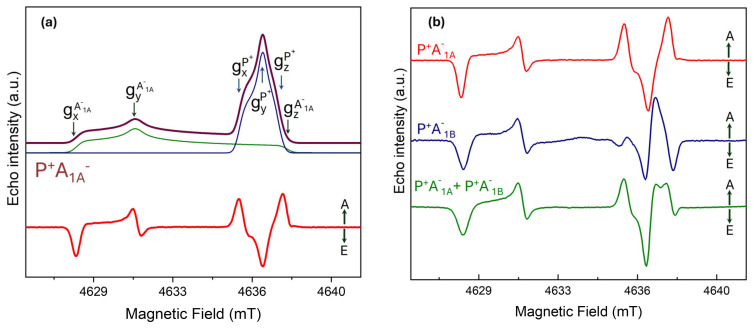
High-frequency (130 GHz) pulsed EPR spectra of the P_700_^+^ A^−^_1_ radical pairs in fully deuterated cyanobacterium, *S. leopoliensis*. (**a**) P_700_^+^A_1A_^−^ pair in thermal equilibrium (top, purple) and in spin-polarized SCRP state (bottom, red) at 100 K. Green and blue spectra are the simulations for EPR signals of A_1A_^−^ and P_700_^+^ in thermal equilibrium, respectively [21]. Positions of the g-tensor main components for A_1A_^−^ and P_700_^+^ are shown by arrows. (**b**) SCRP in A branch, P_700_^+^A_1A_^−^, recorded in “native” PSI (red); SCRP in B branch, P_700_^+^A_1B_^−^, recorded in PSI sample containing sodium hydrosulfite and pre-reduced by illumination at 205–245 K (blue). Photoaccumulation procedure allows reduction of F_A_, F_B_, F_X_, and A_1A_ but not A_1B_: SCRP in both A and B branches (P_700_^+^A_1A_^−^ and P_700_^+^A_1B_^−^ with 1:1 ratio) recorded in PSI where the three [4Fe-4S] clusters, F_X_, F_A_ and F_B_, were biochemically removed to prevent forward ET from the quinones (green). Arrows indicate absorption (A) and emission (E) contributions to the SCRP spectra. T = 100 K, DAF = 1 μs.

Figure 2a shows HF (130 GHz) pulsed EPR spectra of the P_700_^+^A_1A_^−^ radical pair from fully deuterated cyanobacterium *S. leopoliensis* in thermal equilibrium (purple) and in the spin-polarized SCRP state (red) recorded at 100 K. Green and blue spectra in Figure 2a are the simulations for EPR spectra in thermal equilibrium of A_1A_^−^ and P_700_^+^, respectively. The low-field part of the spectra is dominated by the signal from the quinone acceptor A_1_^−^, while the high-field part of the spectra is dominated by signals from the primary donor P_700_^+^. The rhombic g-tensor components g_x_ and g_y_ of A_1A_^−^ are clearly resolved and well-separated from the P_700_^+^ signal. Note that the combination of HF 130 GHz EPR and fully deuterated PSI enables clear resolution of the rhombic g-tensors of both the quinone anion radical A_1A_^−^ and the chlorophyll cation radical P_700_^+^. No hyperfine structure is visible under these conditions. The TR-EPR signal shows the derivative type of lines characteristic of the SCRP spectrum of P_700_^+^A_1_^−^ (Figure 2). It is important to mention that the shape and the phase of the lines in the SCRP depend on the mutual orientation of the g-tensors of P_700_^+^ and A_1_^−^ and thus the respective molecular orientation. As a consequence, the SCRP spectra are sensitive to whether the SCRP in the A or B branches are detected, i.e., the SCRP P_700_^+^A_1A_^−^ and P_700_^+^A_1B_^−^ give different EPR spectra and can thus be distinguished from each other. A detailed technical discussion of this topic is presented in previous publications [23,24,34].

As discussed above, to observe the difference in the line shapes for the transient SCRP P_700_^+^A_1_^-^ from A and B branches, we employed established methods for blocking ET beyond the secondary acceptor A_1_ by (photo)chemical reduction of the later electron acceptors in PSI. The light-induced HF TR-EPR signal observed from the dark-adapted non-reduced deuterated *S. leopoliensis* PSI sample containing the mild reductant sodium ascorbate (Figure 2b, red) is due to ET through the A branch where the ET beyond A_1A_ is blocked at temperatures below 100 K [29], and thus the P_700_^+^A_1A_^−^ SCRP is observed. This SCRP recombines and the next laser flash will again generate the P_700_^+^A_1A_^−^ SCRP. Hence, this SCRP is referred to as “reversible” or “cyclic”. For this type of sample, the SCRP P_700_^+^A_1B_^−^ does not contribute to the spectrum because in this case, ET along the B chain proceeds beyond A_1B_ to generate long-lived or stable P_700_^+^F_X_^−^, P_700_^+^F_A_^−^, and P_700_^+^F_B_^−^ states.

To observe the signature of B branch SCRP, PSI samples containing sodium hydrosulfite were pre-reduced by illumination at 205–245 K and then the temperature lowered to 100 K. This so-called photoaccumulation procedure reduces F_A_, F_B_, F_X_, and A_1A_ but not A_1B_ [55,56]. The light-induced HF TR-EPR spectrum after this treatment is shown in blue in Figure 2b. This is a characteristic spectrum of SCRP in B branch, P_700_^+^A_1B_^−^ (Figure 2b, blue). The different line shapes of the two SCRP spectra of P_700_^+^A_1A_^−^ and P_700_^+^A_1B_^−^, particularly at the high-field portions where P^+^ is dominating, are caused by the different directions of the P_700_^+^-A_1A_^−^ and P_700_^+^-A_1B_^−^ interspin vectors in the g-tensor principal axes system of P_700_^+^. Differences in the A_1_^−^ contribution to the spectrum are much more subtle since the two interspin vectors are very comparable in the g-tensor principal axis system of either quinone. Small changes in the g_x_ value and linewidth of the two quinones have been reported [23,24,34].

SCRPs in both A and B branches can be detected simultaneously in PSI where the three Fe-S clusters (F_X_, F_A_ and F_B_) were removed to prevent forward ET from the quinones to the [4Fe-4S] clusters (Figure 2b, green). In this case, the TR-EPR spectrum at 100 K is composed of two overlapping spectra: one from SCRP in the A branch (P_700_^+^A_1A_^−^), and another one from SCRP formed in the B branch (P_700_^+^A_1B_^−^). This observed spectrum can be modeled as the sum of the SCRP in A and B branches with an almost equal ratio. This is direct evidence that in native PSI from the cyanobacterium *S. leopoliensis* both branches are equally active at low temperature.

**Figure 3:**
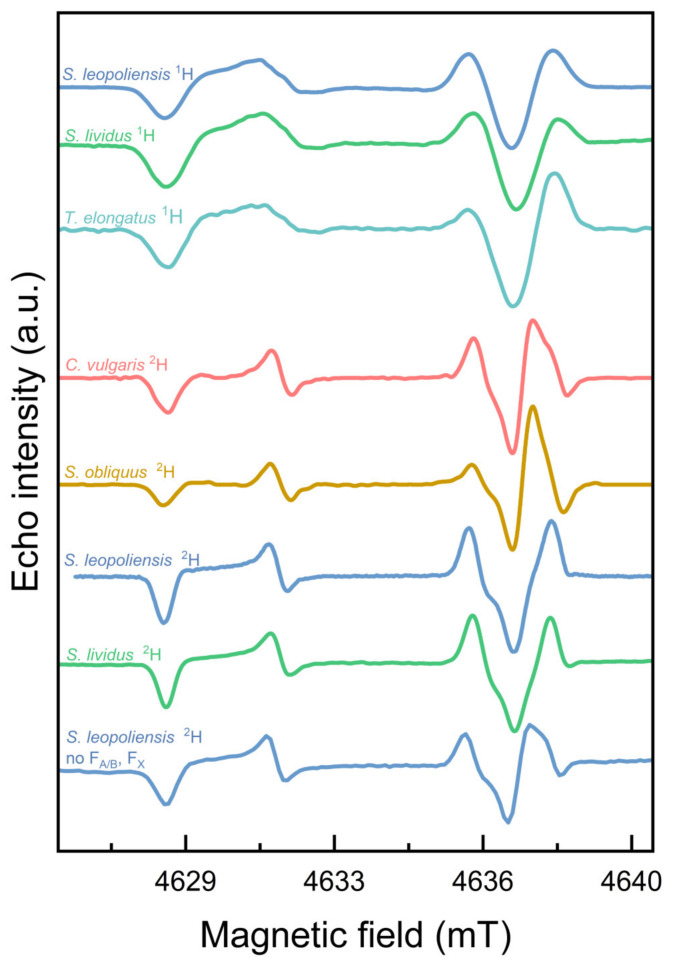
High-frequency D-band (130 GHz) pulsed EPR spectra of the SCRPs recorded in PSI from different biological species. T = 100 K, DAF = 1 μs.

The pulsed HF TR-EPR spectra of SCRPs recorded for five biological species (*S. lividus*, *S. leopoliensis*, *T. vestitus*, *C. vulgaris*, and *S. obliquus*) are shown in Figure 3. All spectra (except some *S. leopoliensis* samples) were recorded on PSI complexes with a small amount of the mild reductant sodium ascorbate added to ensure that in dark-adapted PSI the primary donor is reduced [23,34]. This reductant is not reducing enough to change the oxidation state of the [4Fe-4S] clusters, F_X_, F_A_ and F_B_. When available, spectra were recorded both for fully protonated and fully deuterated PSI.

The SCRP spectra recorded for protonated PSI are very similar, indicating that no major changes in the g-tensors of either P_700_^+^ or A_1_^−^, or in the mutual orientation of these spin-carrying cofactors occurred (Figure 3). Thus, the overall surrounding of these two cofactors and the overall structural arrangement are comparable in all protonated samples from the different species. While there are minor differences in the spectral line shape in the P_700_^+^ region, these differences are hard to interpret. In contrast, clear differences can be observed in the line shapes of SCRP from deuterated proteins, where the linewidth is significantly reduced due to the reduction of hyperfine interaction (Figure 3). In particular, the P_700_^+^ region of the spectra recorded for deuterated PSI are different. The line shape of SCRP spectrum in *S. leopoliensis* and *S. lividus* is typical for A branch SCRP, P_700_^+^A_1A_^−^ (compared with red spectrum in Figure 2b). In the case of *C. vulgaris,* the line shape is different and resembles the line shape of the SCRP in *S. leopoliensis* with F_A_, F_B_, and F_X_ clusters removed, where both SCRPs in A and B branches are visible (Figure 2, green). For the ease of comparison, the SCRP of deuterated *S. leopoliensis* with F_A_, F_B_, and F_X_ clusters removed were recorded under similar experimental conditions and a good agreement with the *C. vulgaris* spectrum was observed (Figure 3). For PSI from *S. obliquus,* the differences of the SCRP with the A branch SCRP are even more pronounced and instead it resembles the blue spectrum shown in Figure 2b, where only SCRP in the B branch, P_700_^+^A_1B_^–^, is observed.

The straightforward interpretation of these data is as follows. In *C. vulgaris* at low temperatures, ET does not proceed beyond acceptor A_1_ in both branches and thus both P_700_^+^A^−^_1A_ and P_700_^+^A^−^_1B_ SCRPs are visible in the TR-EPR spectra in almost equal amounts. In *Scenedesmus obliquus*, only the B branch SCRP P_700_^+^A_1B_^−^ is visible. A plausible explanation is that at low temperatures in *Scenedesmus obliquus* ET beyond A_1_ is blocked in the B branch but can proceed to the Fe-S cluster via the A branch. These results indicate that low temperature ET pathways in green algae PSI are different from those in cyanobacteria. In previous studies, differences in the directionality of electron ET between prokaryotic and eukaryotic PSIs were observed, with the fraction of the B branch generally being larger and having a longer lifetime in eukaryotic PSI compared to prokaryotic PSI [57].

**Scheme 1:**
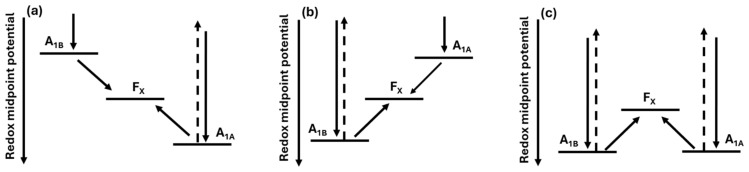
Schematic presentation of relative redox potential of acceptors A_1A_ and A_1B_ with respect to F_x_. Note that lower redox midpoint potential means more reducing potential. Dashed arrow indicates recombination reaction to P_700_^+^. (**a**) Cyclic electron transfer in the A branch. (**b**) Cyclic electron transfer in the B branch. (**c**) Cyclic electron transfer in both A and B branches.

The difference of the low temperature ET in PSI and relative activity of A and B branches was explained by relative redox potential of acceptors A_1A_ and A_1B_ in respect to F_x_ (see Scheme 1). If the redox potential of A_1A_ is higher (less reducing) compared to the redox potential of F_x_, then at low temperatures cyclic ET is observed in A branch. If redox potential of A_1B_ is higher (less reducing) than the redox potential of F_x_, then ET in B branch is cyclic/reversable. In the case when F_x_ potential is lower than redox potential of both acceptors A_1A_ and A_1B_, cyclic ET can be observed in both branches with the ratio determined by relative potential of A_1A_ to A_1B_ [38,40].

Importantly, in 1999, Joliot and Joliot observed at ambient temperature biphasic kinetics of oxidation of reduced phylloquinone and attributed these two phases to oxidation, A_1A_ and A_1B_, respectively. Since the amplitudes of both phases were comparable, they interpreted this as a sign of equal ET activity of both branches in green algae *Chlorella sorokiniana* [58]. This is an indication of the similar microenvironment and, as a consequence, similar redox potential of A_1A_ and A_1B_ acceptors. At low temperature this might result in cyclic/reversible ET in both the A and B branches in *Chlorella sorokiniana* PSI, similar to what we observed for *C. vulgaris*.

### 2.2. Iron Sulfur Clusters

**Figure 4:**
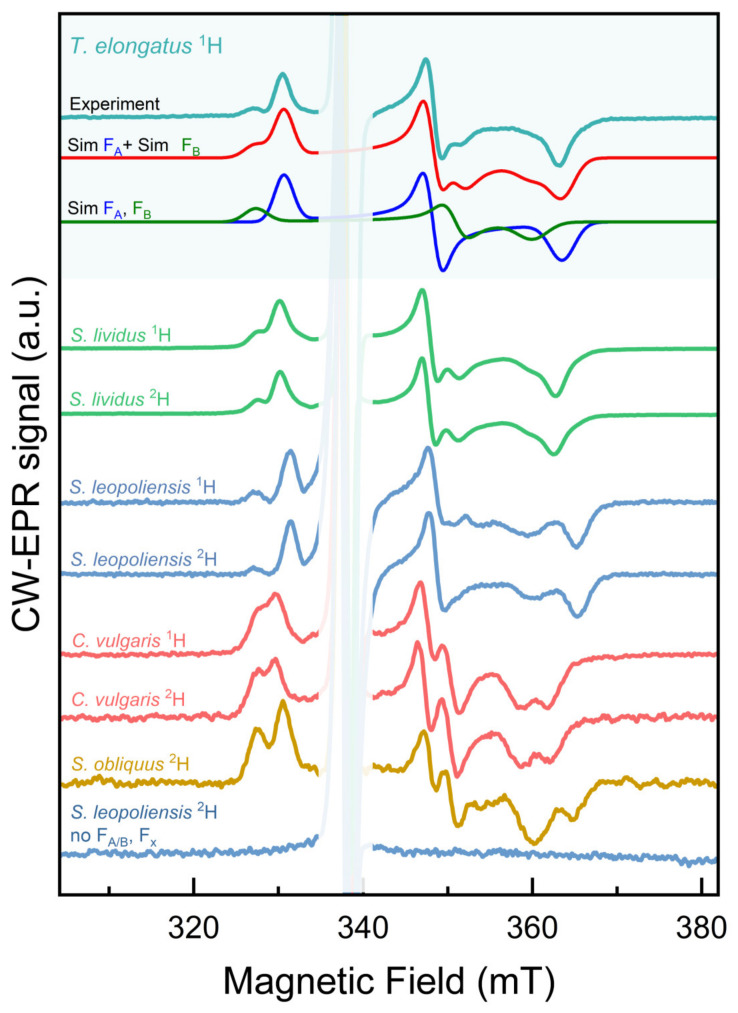
Continuous wave (cw) X-band (9.5 GHz) EPR spectra of various PSI samples. Samples were frozen in the dark, cooled down to 10 K in the cavity of the EPR spectrometer, and subsequently illuminated with a white light LED. Microwave power, 3 mW; modulation amplitude, 1.2 mT.

Iron sulfur [4Fe-4S] clusters within PSI play a vital role in ET. The three [4Fe-4S] clusters (F_X_, F_A_ and F_B_) shuttle electrons from the site of initial charge separation to the stromal side where mobile electron transfer proteins ferredoxin or flavodoxin are facilitating further ET [10,59,60,61]. As pointed out above, the relative redox potential of A_1A_ and A_1B_ in respect to F_X_ determines whether one or another branch of ET in PSI is active at low temperatures and photoinduced electron transfer can proceed through a quinone acceptor to the final acceptors. EPR spectroscopy is a highly informative technique for detecting and resolving signals from individual [4Fe-4S] clusters, offering structural insights otherwise challenging to obtain [10,62,63,64,65,66]. To ascertain if there are substantial differences between electronic characteristics of [4Fe-4S] complexes in the proteins under study and test the relationships between these properties and directionality of ET, we analyzed EPR spectra of the reduced [4Fe-4S] clusters. The cw X-band EPR spectra of the reduced [4Fe-4S] clusters F_A_ and F_B_ (Figure 4) were recorded for the same five biological species as above: *S. lividus*, *S. leopoliensis*, *T. elongatus*, *C. vulgaris*, and *S. obliquus*.

In the oxidized state, the [4Fe-4S]^2+^ are diamagnetic and thus give no EPR signal. In the reduced state, [4Fe-4S]^1+^, one ferric and three ferrous iron atoms within the clusters are magnetically coupled, resulting in an effective total spin of S = ½ [10]. EPR signals from [4Fe-4S] clusters have extremely short relaxation times; thus, the measurements were performed at 10 K. Freezing a PSI complex in the dark, with subsequent illumination at low temperature allows promotion of only one electron from P_700_ to either F_A_ or F_B_, but not both, in a given PSI complex. Consequently, the resulting EPR spectra represent a sum of signals from reduced centers with three distinct g-values, reflecting ”rhombic” symmetry of the g-tensor. Under our experimental conditions, signals of the [4Fe-4S] cluster F_X_ cannot be observed. To obtain g-tensor parameters g_x_, g_y_, and g_z_ of both F_A_ and F_B_ centers and their relative contribution to the experimental spectra (F_A_/F_B_ ratio), the spectra were simulated (see Figure S1, Table S1). Selected simulation parameters are summarized in Table 1.

The g-tensor principal values are in the typical range for reduced F_A_ and F_B_ clusters reported in the literature. While these values are slightly different for each type of PSI, there is no clear trend between the species. Within the experimental error, no differences in the g-values or F_A_/F_B_ ratio between protonated and deuterated proteins were detected.

We observed that the F_A_/F_B_ ratios in PSI of green algae differ from those of PSI complexes from cyanobacteria. For the thermophilic cyanobacteria *T. elongatus* and *S. lividus*, the F_A_ to F_B_ ratio is between 2 and 2.7. In the mesophilic *S. leopoliensis*, this ratio increases to 3.6. In contrast, for the two green algae, *C. vulgaris* and *S. obliquus*, the F_A_ to F_B_ ratio is approximately equal. This can be correlated to the activity of A or B branches at low temperatures and, as a consequence, to the redox potential of A_1A_ and A_1B_ relative to F_X_.

Indeed, in *T. elongatus*, *S. lividus*, and *S. leopoliensis*, the F_A_/F_B_ ratio exceeds one. In these proteins, low temperature ET is cyclic in the A branch and irreversible in the B branch. This can be explained by the following order of the PSI acceptors’ redox potential: A_1A_ > F_x_> A_1B_. The observed ratio F_A_/F_B_ of 2 and 3.6 can be accounted for by a slightly higher redox potential of F_A_. On the contrary, in *C. vulgaris* and *S. obliquus*, where the F_A_/F_B_ ratio is close to one, the B branch is more active in cyclic ET at low temperature. In *S. vulgaris,* the cyclic ET was observed in both A and B branches, indicating that their redox potential is higher than the redox potential of F_X_. In *S. obliquus, only* B branch demonstrates cyclic ET. Equal contribution of F_A_ and F_B_ to the EPR spectrum can be explained by equal redox potential of these complexes.

## 3. Materials and Methods

### 3.1. Sample Preparation

***Synechococcus lividus* PSI**. PSI RCs were prepared from whole cells of the cyanobacterium *S. lividus* as described previously [67]. The final buffer was 20 mM HEPES, pH 8, 0.03% β-DM (n-dodecyl-β-D-maltopyranoside, Anatrace). Sodium ascorbate was added to a final concentration of 5 mM from a concentrated stock solution of 0.64 M sodium ascorbate in 50 mM MES, pH 6. For EPR measurements, PSI was concentrated with 50 kDa MWCO microconcentrators (Millipore, Burlington, MA, USA) to the desired concentration. All samples were kept in the dark and on ice until used for EPR.

***Synechococcus leopoliensis* PSI.** PSI RCs were isolated from whole cells of the cyanobacterium *S. leopoliensis* which were grown either in H_2_O or in D_2_O (99.6%). Purified PSI was prepared in 20 mM HEPES, pH 8, and 0.03% β-DM. Sodium ascorbate (5 mM) was added prior to EPR measurement. The [4Fe-4S] clusters (F_X_, F_A_, F_B_) were removed by established preparations. PSI (0.2 mg of chlorophyll/mL) was incubated in a buffer containing 6.8 M urea, 62 mM Tris, and 76 mM glycine–NaOH, pH 10, for 1 h, to remove F_A_/F_B_ as previously described [68]. The PSI sample was then dialyzed overnight against 50 mM Tris-HCl, pH 8.3. To remove F_X_, the sample was further treated with 3 M urea, 5 mM K3FeCN6, and 50 mM Tris-HCl, pH 8.0, for 4.5 h [69]. The PSI sample was dialyzed overnight against 50 mM Tris-HCl, pH 8.3, and 5 mM 4,5-dihydroxy-1,3-benzene disulfonic acid (disodium salt) and then again overnight against two changes of 50 mM Tris-HCl, pH 8.0, 0.03% β-DM. The sample, analyzed by ICP-AES (ThermoFisher Scientific, Waltham, MA, USA), showed a ratio of ∼1 Fe/PSI monomer after urea treatment, confirming quantitative removal of the three [4Fe-4S] clusters. For EPR measurements, 5 mM sodium ascorbate or 50 mM Tricine-NaOH, pH 8.0, and 10 mM sodium hydrosulfite were added prior to freezing in liquid nitrogen.

***Thermosynechococcus elongatus* PSI** (previously named *Synechococcus elongatus and* recently renamed *T. vestitus*). Trimeric photosystem I was extracted from whole cells of the cyanobacterium *T. elongatus* which were cultivated as previously described [70]. After separating PSI from other solubilized protein using a DEAE650 anion exchanger column (Tosoh Bioscience LLC, Grove City, OH, USA), the PSI fractions were pooled and purified using a SP Sepharose column with buffer A (5 mM Mes, pH 5.5; 0.013% C12E8 (octaethyleneglycol monododecyl ether, Anatrace)) and B (5 mM Mes, pH5.5; 500 mM NaCl; 0.013% C12E8). The purified PSI fractions were pooled again and washed with storage buffer containing 5 mM Mes, pH 6; 30 mM MgSO_4_, and 0.013% C12E8 using an Amicon concentrator (Amicon, Miami, FL, USA) with 100 kDa cutoff. Once a concentration of 7.5 mM chlorophyll was reached, trimeric PSI was diluted with buffer containing 5 mM MES pH 6 and 0.013% C12E8 to start crystallization. The crystallization took place overnight and resulted in rectangular shaped crystals of about 40 μm in the longest dimension. Afterward, the crystals were pelleted, and after removal of the supernatant, storage buffer was added to dissolve the crystals and yield a solution of PSI at the desired chlorophyll concentration.

***Chlorella vulgaris* and *Scenedesmus obliquus* thylakoid preparation.** *Chlorella vugaris* and *Scenedesmus obliquus* were grown in 99.6% heavy water as described [71]. Algal cells (5 g) were resuspended in 30 mM Tricine-NaOH, pH 8.0, 300 mM sucrose, and 15 mM NaCl. The cell suspension was placed in a pre-chilled Bead-Beater (BioSpec Products, Inc., Bartlesville, OK, USA) with 1 mm glass beads. The sample was beaten for 5 × 1 min bursts, with 5 min rest in between with cooling in a surrounding ice bath. The solution was decanted and spun at 2000 rpm for 2 min in a Beckman Coulter Avanti J-26 XP with a JLA 16.25 rotor (Beckman Coulter, Brea, CA, USA) to remove glass beads. Unbroken cells and starch were removed by centrifugation at 7000 rpm for 10 min in the JLA 16.25 rotor. The supernatant was spun at 45,000 rpm for 2 h in a Beckman L-60 ultrafuge with a 60 Ti rotor. The pellets were resuspended in 30 mM Tricine-NaOH, pH 8.0, 300 mM sucrose, and 150 mM NaCl and incubated on ice for 30 min. The sample was pelleted by ultracentrifugation at 45,000 rpm for 2 h. The pellet was resuspended in 30 mM Tricine-NaOH, pH 8.0, 300 mM sucrose, and 15 mM NaCl at a concentration ~2 mg/mL Chl and stored at −80 °C.

***Chlorella vulgaris* and *Scenedesmus obliquus* PSI isolation.** PSI was extracted from thylakoid membranes diluted to 1 mg/mL Chl by addition of 2% β-DM. Following 30 min incubation on ice, insoluble material was removed by centrifugation at 45,000 for 30 min in a Beckman L-60 ultrafuge with a 60 Ti rotor. The supernatant was loaded onto a Toyopearl DEAE 650-C column equilibrated with 30 mM Tricine, pH 8, 15 mM NaCl and 0.2% β-DM. Protein was eluted from the column with a linear NaCl gradient (15–250 NaCl) in 30 mM Tricine-NaOH pH 8.0, 0.2% β-DM. The middle dark green fractions were pooled and precipitated with 10% PEG3350. The sample was immediately centrifuged at 5000 rpm in a Beckman Coulter Avanti JA30.50 rotor for 5 min. The green pellet was resuspended in 30 mM Tricine-NaOH, pH 8.0, 0.05% β-DM. The sample was loaded onto a 15–40% sucrose density gradient prepared in 30 mM Tricine-NaOH, pH 8.0, 0.05% β-DM and 15 mM NaCl and spun overnight at 40,000 rpm in a 50Ti rotor at 4 °C. The lower band containing PSI was collected. The upper green band contained LHCs. The PSI was repeatedly washed to remove the sucrose using 50 kDa MWCO microconcentrators (Millipore). PSI was stored at −80 °C until thawed for EPR use. Samples were further concentrated, and 5 mM sodium ascorbate was added prior to EPR measurements.

### 3.2. EPR Spectroscopy

**X-band:** Continuous wave (cw) X-band (9.5 GHz) EPR measurements were carried out with a Bruker ELEXSYS II E500 EPR spectrometer (Bruker Biospin Corp, Ettlingen, Germany) equipped with a TE_102_ rectangular EPR resonator (Bruker ER 4102ST) and helium gas-flow cryostat (ICE Oxford, Witney, UK). Temperature control was provided by an ITC (Oxford Instruments, Abingdon, UK). The cw EPR experiments used field modulation with phase sensitive lock-in detection. This type of detection results in the first derivative-type EPR spectra. EPR samples were prepared in a N_2_ box, placed in 4 mm o.d. quartz EPR tubes, capped, and frozen under dark conditions in liquid N_2_ prior to placement in the pre-cooled EPR resonator. Illumination of the sample was achieved with a Solis-3c “Day Light White” LED (Thorlabs, Newton, NJ, USA). The spectra of the Fe-S clusters were recorded at low temperature before and after illumination and the dark spectrum before illumination was subtracted.

**D-band:** EPR measurements were performed on a pulsed/continuous-wave high-frequency (HF) D-band (130 GHz/4.6 T) EPR spectrometer [27,72] with a single mode TE_011_ cylindrical resonator. Pulsed EPR spectra of stable radical species were recorded by monitoring the electron spin echo (ESE) intensity from a two-microwave pulse sequence as a function of magnetic field. Pulsed TR-EPR spectra were recorded in a similar way by initial photoexcitation of the protein by a short (<10 ns) laser pulse followed by the microwave pulses (π/2−τ−π−τ−echo) at a fixed delay after flash (DAF) time. The duration of the π/2 microwave pulse was 40–60 ns. Light excitation of the sample was achieved with an optical parametric oscillator (OPO; basiScan, GWU-Lasertechnik, Erftstadt, Germany) pumped by an Nd: YAG laser (Quanta-Ray INDI, Spectra-Physics, Milpitas, CA, USA), the output of which was coupled to an optical fiber. The optical fiber allows delivery of up to 1 mJ/pulse to the sample. The excitation wavelength was 550 nm. The samples were loaded into quartz tubes, dark-adapted, and placed in the precooled microwave cavity. The cavity was mounted in an Oxford Instruments flow cryostat, and temperature was controlled by an Oxford Instruments temperature control system (ITC503). SCRP spectra were then recorded under consistent illumination conditions, 1 μs after the laser flash, and the “dark” spectrum recorded 25 ms after the laser flash was subtracted.

**Data analysis and simulations:** Analysis of EPR spectra was accomplished using EasySpin [73] version 6.0.0 within MATLAB R2023a (The MathWorks, Natick, MA, USA) environment. Continuous wave X-band EPR spectra of reduced [4Fe-4S] clusters F_A_ and F_B_ were simulated as S = ½ systems with rhombic g-tensor and anisotropic line widths.

## 4. Conclusions

EPR spectroscopy was used to study light-induced charge separation in PSI isolated from both prokaryotes (cyanobacteria) and eukaryotes (green algae). In PSI, primary charge separation occurs through two symmetric branches of redox cofactors, the A and B branch, and the bidirectional ET asymmetry can be differentiated with low temperature high-frequency TR-EPR spectroscopy of deuterated PSI complexes. In this work, fully deuterated green algal PSI was utilized to resolve, for the first time, the SCRPs P_700_^+^A_1A_^−^ and P_700_^+^A_1B_^−^ involved in eukaryotic PSI charge separation. Whereas at low temperatures prokaryotic PSI shows cyclic ET in A branch and irreversible ET in the B branch, green algal PSI shows cyclic ET in both A and B branches or only in the B branch. Prior to this study, B branch SCRP P_700_^+^A_1B_^−^ had been observed for “special” PSI mutants and cyanobacterial PSI pretreated with strong reducing conditions or upon removal of the [4Fe-4S] clusters F_X_, F_A_, and F_B_. Light-driven low temperature charge separation from the primary donor P_700_ to F_A_ and F_B_ shows that these clusters are intact in the isolated green algal PSI. Furthermore, we observed a notable species-dependent correlation between low temperature charge separation in the measured F_A_/F_B_ ratio. As the core molecular cofactor ET chains are the same in eukaryotic and prokaryotic PSI structures, we hypothesize that the dynamic protein matrix plays a role in controlling the relative ratios of A and B branch primary charge separation and charge separation to the [4Fe-4S] clusters. These differences could be reflective of localized heterogeneous protein environments or, perhaps, reflect overall protein oligomeric structures, with algal PSI typically being monomeric and attached to multiple LHCI antenna in PSI-supercomplexes versus cyanobacterial PSI which is trimeric and can be purified without bound LHCI. Though further research is necessary to elucidate the underlying mechanisms to explain these experimental variations in PSI charge separation, this study sets the stage for future investigations into the complex interplay between protein structure, ET pathways, and the environmental species-dependent adaptations of photosynthetic organisms.

## Figures and Tables

**Table 1:** g-values of the [4Fe-4S] cluster F_A_ and F_B_, and their relative contribution to the EPR spectrum F_A_/F_B_ as obtained from simulations.

	F_A_			F_B_			F_A_/F_B_
	g_x_	g_y_	g_z_	g_x_	g_y_	g_z_	
***T. elongatus*** **^1^H**	2.048	1.945	1.863	2.069	1.930	1.881	2.7
***S. lividus*** **^1^H**	2.050	1.946	1.865	2.068	1.933	1.879	2.0
***S. lividus*** **^2^H**	2.050	1.947	1.867	2.069	1.933	1.879	2.7
***S. leopoliensis*** **^1^H**	2.044	1.942	1.854	2.070	1.921	1.880	3.6
***S. leopoliensis*** **^2^H**	2.044	1.942	1.854	2.070	1.921	1.880	3.6
***C. vulgaris*** **^1^H**	2.052	1.948	1.869	2.067	1.933	1.886	1.0
***C. vulgaris*** **^2^H**	2.054	1.949	1.868	2.068	1.934	1.886	0.9
***S. obliquus*** **^2^H**	2.046	1.945	1.853	2.067	1.932	1.880	1.3

## Data Availability

Data are contained within the article.

## Supplementary Materials

The following supporting information can be downloaded at: https://www.mdpi.com/article/10.3390/ijms25158188/s1. Figure S1: Experimental and simulated continuous wave (cw) X-band (9.5 GHz) EPR spectra of iron sulfur clusters, F_A_ and F_B_ in various Photosystem I samples, recorded after illumination at 10 K. Table S1: Simulation parameters of the [4Fe-4S] cluster F_A_ and F_B_, and their relative contribution to the EPR spectrum FA/FB (errors vary between species but do not exceed 30%).

## Abbreviations

RC—reaction center; PSI—Photosystem I; PSII—Photosystem II; HF—high frequency; ET—electron transfer; SCRPs—spin correlated radical pairs; EPR—electron paramagnetic resonance; ESE—electron spin echo; DAF—delay after flash.
