# Supplementary material for: Light-Induced Charge Separation in Photosystem I from Different Biological Species Characterized by Multifrequency Electron Paramagnetic Resonance Spectroscopy
Source: Int J Mol Sci. 2024 Jul 26;25(15):8188. doi: 10.3390/ijms25158188 (PMC11311511; doi:10.3390/ijms25158188)
Supplement: Supplementary file 1 [file ijms-25-08188-s001.zip › ijms-3083548-supplementary.pdf]

Supporting Information

for

Light-Induced Charge Separation in Photosystem I from Different Biological Species Characterized by Multifrequency EPR

Jasleen K. Bindra[1], Tirupathi Malavath[1], Mandefro Teferi[1], Moritz Kretzschmar[2], Jan Kern[2], Jens Niklas[1], Lisa M. Utschig[1]*, Oleg G. Poluektov[1]*

[1] Chemical Sciences and Engineering Division, Argonne National Laboratory, 9700 South Cass Avenue, Lemont, Illinois 60439, United States

[2] Molecular Biophysics and Integrated Bioimaging Division, Lawrence Berkeley National Laboratory, Berkeley, California 94720, United States

*Corresponding authors: oleg@anl.gov, Utschig@anl.gov

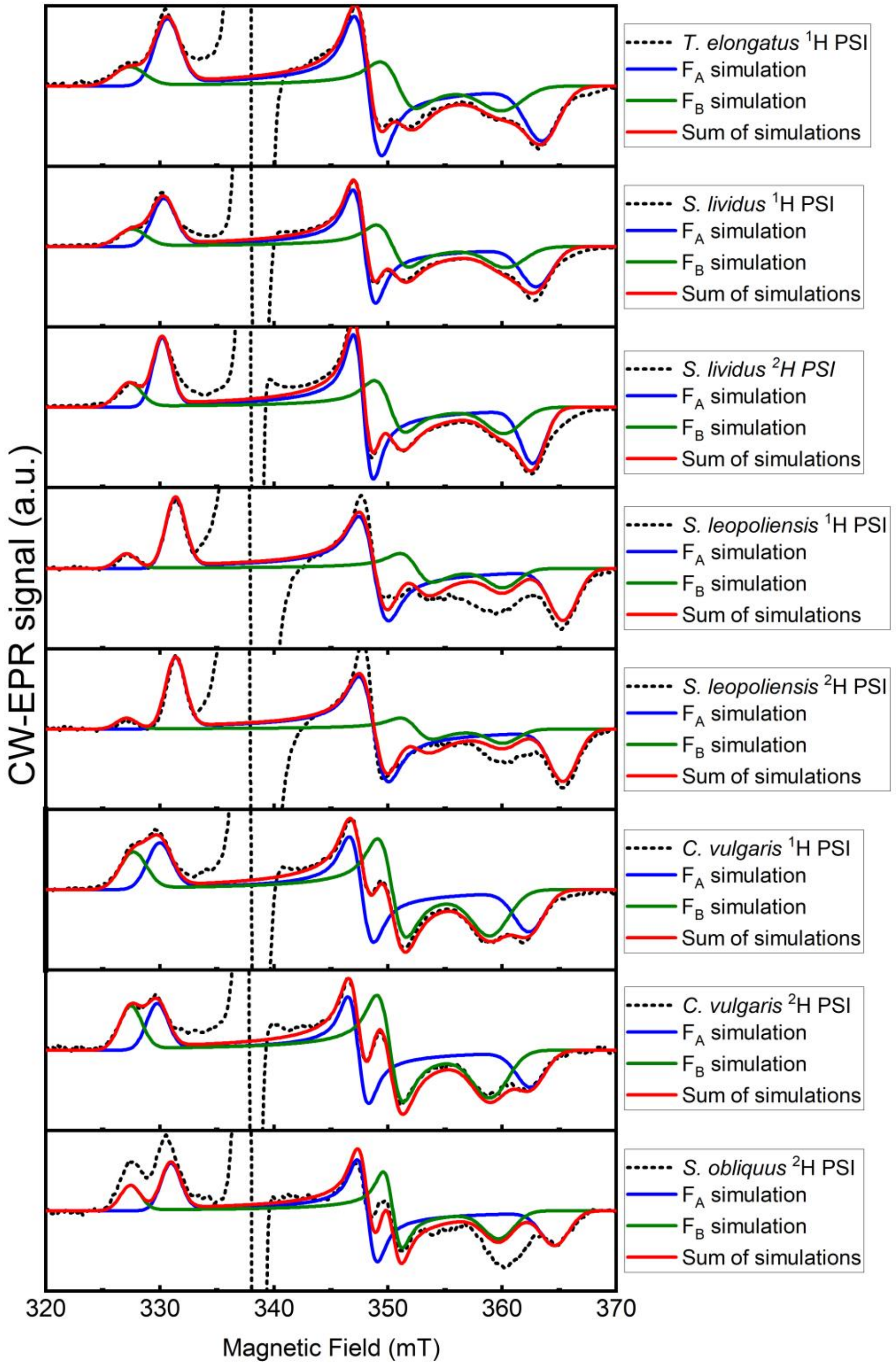

Figure S1: Experimental and simulated continuous wave (cw) X-band (9.5 GHz) EPR spectra of iron sulfur clusters, F_A and F_B in various Photosystem I samples, recorded after illumination at 10 K.

Sample	F_A						F_B						F_A/F_B
	g_x	g_y	g_z	H_x (MHz)	H_y (MHz)	H_z (MHz)	g_x	g_y	g_z	H_x (MHz)	H_y (MHz)	H_z (MHz)	
T. elongatus ^1H PSI	2.048	1.945	1.863	75	59	89	2.069	1.930	1.881	91	77	107	2.7
S. lividus ^1H PSI	2.050	1.946	1.865	70	49	82	2.068	1.933	1.879	84	69	100	2.0
S. lividus ^2H PSI	2.050	1.947	1.867	55	43	71	2.069	1.933	1.879	71	67	94	2.7
S. leopoliensis ^1H PSI	2.044	1.942	1.854	58	64	74	2.070	1.921	1.880	60	70	80	3.6
S. leopoliensis ^2H PSI	2.044	1.942	1.854	58	64	74	2.070	1.921	1.880	60	90	80	3.6
C. vulgaris ^1H PSI	2.052	1.948	1.869	72	53	80	2.067	1.933	1.886	78	62	97	1.0
C. vulgaris ^2H PSI	2.054	1.949	1.868	63	45	77	2.068	11.934	1.886	67	57	94	0.9
S. Obliquus ^2H PSI	2.046	1.945	1.853	58	44	77	2.067	1.932	1.880	60	41	80	1.3

Table S1: Simulation parameters of the [4Fe-4S] cluster F_A and F_B, and their relative contribution to the EPR spectrum FA/FB (errors vary between species but do not exceed 30%).
